# Effect of Anticancer Quinones on Reactive Oxygen Production by Adult Rat Heart Myocytes

**DOI:** 10.1155/2020/8877100

**Published:** 2020-10-22

**Authors:** James H. Doroshow

**Affiliations:** ^1^Division of Cancer Treatment and Diagnosis, National Cancer Institute, NIH, Bethesda, MD 20892, USA; ^2^Developmental Therapeutics Branch of the Center for Cancer Research, National Cancer Institute, NIH, Bethesda, MD 20892, USA; ^3^Department of Medical Oncology and Therapeutics Research, City of Hope Comprehensive Cancer Center, Duarte, CA 91010, USA

## Abstract

This study investigated the effect of anthracycline antibiotics, mitomycin C, and menadione on oxygen consumption and hydrogen peroxide production by intact, beating, rat heart myocytes. Doxorubicin produced a dose-dependent increase in the rate of cyanide-resistant respiration by beating myocytes. The anthracycline analogs 4-demethoxydaunorubicin, 4′-epidoxorubicin, 4′-deoxydoxorubicin, and menogaril, as well as the anticancer quinones mitomycin C and menadione, also significantly increased oxygen consumption by cardiac myocytes. However, 5-iminodaunorubicin (which has a substituted quinone group) and mitoxantrone (which is not easily reduced by flavin dehydrogenases) had no effect on cardiac respiration. Both catalase (43%) and acetylated cytochrome c (19%) significantly decreased oxygen consumption that had been stimulated by doxorubicin; furthermore, extracellular hydrogen peroxide production was increased from undetectable control levels to 1.30 ± 0.02 nmol/min/10^7^ myocytes (*n* = 4, *P* < 0.01) in the presence of 400 *μ*M doxorubicin. These experiments suggest that the anthracycline antibiotics and other anticancer quinones stimulate cardiac oxygen radical production in intact heart myocytes; such a free radical cascade could contribute to the cardiac toxicity of these drugs.

## 1. Introduction

The anticancer quinones, including the anthracycline antibiotics and mitomycin C, are widely used for the treatment of hematopoietic malignancies and cancers of the breast and bladder [1]. Unfortunately, the utility of these drugs is hindered by cardiac toxicity that most often takes the form of a dose-related congestive cardiomyopathy [2–4]. Many different hypotheses have been suggested to explain the myocardial injury produced by antineoplastic quinones [5, 6]. These hypotheses include the observation that the formation of a doxorubicin-iron complex may enhance the formation of strong oxidants toxic to the heart [7]; cardiac toxicity could also occur through drug-induced effects on iron-binding proteins [8]. However, several lines of evidence support the possibility that stimulation of superoxide anion, hydrogen peroxide, and a chemical species with the characteristics of the hydroxyl radical in the heart after reduction of the quinone moiety by complex I of the mitochondrial electron transport chain [9], NADPH:cytochrome P-450 reductase associated with the sarcoplasmic reticular membrane, or cytoplasmic xanthine dehydrogenase [10] plays an important role in the mechanism of anthracycline cardiac toxicity [11, 12].

If reactive oxygen species are produced at these intracellular sites in intact heart cells [13], oxidative stress itself might explain the characteristic picture of mitochondrial swelling, sarcotubular vacuolization, and myofibrillar loss produced by anthracycline quinones [1]. Previous investigations utilizing neonatal rat heart cells in culture have demonstrated that treatment with the anthracycline analog daunorubicin decreases cellular reduced glutathione pools [14] and that inhibition of glutathione-mediated peroxide detoxification significantly enhances the cytotoxicity of doxorubicin [15]. Hence, drug-induced oxygen radical formation appears to be at least one mechanism involved in the toxicity of anthracyclines for neonatal heart cells.

To confirm previous studies that used cardiac subcellular fractions from adult rats (but without disrupting intracellular architecture or utilizing cells that are actively dividing) [10], the experiments presented here were performed with intact, actively beating myocytes from adult rats. Our results indicate that the anthracycline and mitomycin C quinones are reduced by one electron in adult rat heart cells and can initiate an oxidation-reduction cycle in the presence of molecular oxygen that ultimately results in the formation of hydrogen peroxide.

## 2. Materials and Methods

### 2.1. Experimental Animals

Male 200-gram Sprague-Dawley rats were obtained from Simonsen Laboratories, Gilroy, CA and housed on hardwood bedding with access to feed and water *ad libitum*.

### 2.2. Materials

Doxorubicin hydrochloride and mitomycin C were purchased from commercial sources. Menogaril, 5-iminodaunorubicin, and mitoxantrone were supplied by the Drug Synthesis and Chemistry Branch, Division of Cancer Treatment and Diagnosis, National Cancer Institute, Bethesda, MD. 4-demethoxydaunorubicin, 4′-epidoxorubicin, and 4′-deoxydoxorubicin were a gift of Farmitalia Carlo Erba, Milan, Italy. Menadione (2-methyl-1,4-naphthoquinone, sodium bisulfite salt), cytochrome c (type VI from horse heart), dimethyl sulfoxide (DMS0), diethylenetriaminepentaacetic acid (DTPA), hyaluronidase (type I-S), and bovine erythrocyte superoxide dismutase (SOD, 2900 units/mg) were from Sigma-Aldrich Chemical Co., St. Louis, MO. Prior to these experiments, cytochrome c was acetylated to eliminate the effects of potential exogenous oxidizing or reducing species present in our reaction mixtures [16]. Collagenase (type II) was purchased from Cooper Biomedical Inc., Freehold, NJ. Metrizamide (type AN 6300) of analytical grade was from Accurate Chemical and Scientific Corp., Westbury, NY. Catalase of analytical grade (65,000 units/mg) was purchased from Boehringer Mannheim Biochemicals, Indianapolis, IN, and was devoid of SOD activity when assayed by the method of McCord and Fridovich [17]. Only glass distilled, deionized water was used in these studies.

### 2.3. Preparation of Cardiac Myocytes

Experimental animals were euthanized by lethal inhalation of methoxyflurane. Ventricular tissue, typically from 6-8 rats, was rapidly removed and then washed and minced in an iced phosphate buffer at pH 7.4 containing 120 mM NaCl, 5.4 mM KCl, 1.5 mM Na_2_HP0_4_, 0.4 mM NaH_2_P0_4_, and 5 mM glucose. Myocytes were prepared as previously described [18] except that separation of intact from damaged cells was performed by metrizamide density centrifugation [19]. Cell viability in these studies was determined by the presence of rod-shaped morphology and exclusion of 0.1% trypan blue dye and ranged in typical experiments from 70-80%; myocyte yield was usually 0.5 to 1 × 10^6^ viable cells per heart.

### 2.4. Measurement of Oxygen Consumption and Hydrogen Peroxide Formation

Oxygen consumption was determined using a YSI oxygen monitor with a Clarke-type electrode as previously described [20]. The final, 3 ml reaction mixture contained 125 mM potassium phosphate, 140 mM sodium chloride, and 10 mM glucose, pH 7.4 at 37°C. All reactants were bubbled with air for 30 min at 37°C before use; and most experiments were performed with 2 × 10^6^ viable myocytes in the final 3 ml reaction mixture. Where utilized, myocytes were preincubated with 5 mM KCN for 10 min in the stirred, temperature-controlled glass oxygen electrode chamber open to the atmosphere prior to the initiation of the reaction with a chemotherapeutic agent. After insertion of the oxygen electrode, the linear rate of oxygen consumption was determined for 10 to 15 min thereafter. In some experiments, small volumes of specific reagents (typically 10 *μ*l of catalase) were added to the reaction vessel through the access slot of the oxygen electrode plunger. The rate of oxygen consumption was based on a value of 597 nmol for the total dissolved oxygen content of the reaction vessel [21]. Hydrogen peroxide production was quantitated by the release of oxygen into the closed reaction chamber of the oxygen electrode as previously described [22], after the addition of 7500 units of catalase through the access slot of the plunger.

### 2.5. Statistical Methods

Data were analyzed with the 2-tailed *t*-test for independent means (not significant, *P* > 0.05 [23]).

## 3. Results

### 3.1. Effect of Anticancer Quinones on Oxygen Consumption by Rat Heart Myocytes

Oxygen consumption by intact, untreated cardiac myocytes isolated from adult rats was (mean ± S.E.) 5.35 ± 0.38 nmol/min/2 × 10^6^ cells (Table 1). Cardiac respiration was reduced by 81% after the addition of KCN to inhibit mitochondrial cytochrome oxidase. Treatment with rotenone to inhibit mitochondrial electron flow beyond complex I of the electron transport chain decreased oxygen consumption by 72%. As demonstrated in Table 1, in the presence of myocytes, doxorubicin significantly increased the rate of both rotenone-resistant and cyanide-resistant respiration. In analogy to our previous experiments with cardiac submitochondrial particles [9] and cardiac sarcoplasmic reticulum [10], these studies suggest that the doxorubicin quinone may be reduced by mitochondrial complex I or other intracellular flavin dehydrogenases with subsequent electron transfer to molecular oxygen in the heart. The difference between our results with rotenone and KCN may reflect the inhibition of myocardial copper-zinc superoxide dismutase by cyanide. This could decrease the enzymatic breakdown of intracellular superoxide (ultimately to O_2_ and H_2_O) leading to an apparent increase in respiratory rate.

**Figure 1:**
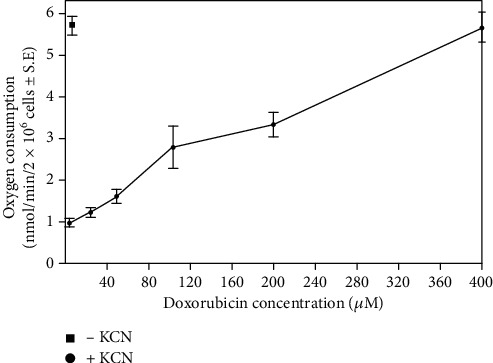
Effect of doxorubicin concentration on the rate of cyanide-resistant respiration in adult rat heart myocytes. Data represent the mean ± S.E. of 3 to 6 experiments for every concentration of doxorubicin tested; *P* < 0.05 for each drug level compared to the untreated control.

**Figure 2:**
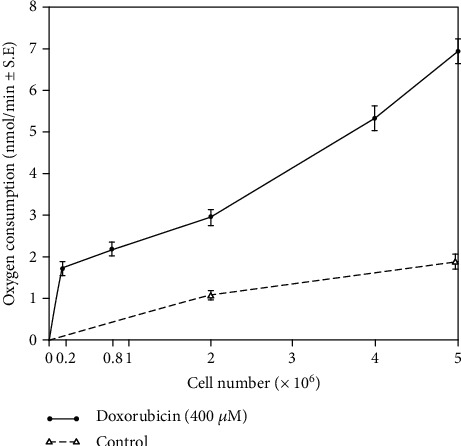
Effect of myocyte concentration on doxorubicin-stimulated, cyanide-resistant (5 mM) oxygen consumption. The results shown represent the mean ± S.E. of 3 experiments for each myocyte concentration tested.

In the presence of cyanide, doxorubicin enhanced the respiratory rate of adult cardiac myocytes in a concentration-dependent manner (Figure 1); a significant increase in oxygen consumption occurred under our experimental conditions beginning with a doxorubicin concentration of 10 *μ*M, (1.4 ± 0.01 nmol/min/2 × 10^6^ cells, *P* < 0.05, *n* = 3). At the highest doxorubicin concentration tested (400 *μ*M), drug-stimulated oxygen consumption was equivalent to that produced intrinsically by mitochondrial respiration. Furthermore, as shown in Figure 2, drug-stimulated oxygen consumption varied with the myocyte concentration examined. No oxygen was consumed in the absence of cells, and the difference in respiratory rate between drug-treated and control cells increased with the number of myocytes studied (Figure 2).

We also investigated the effect of other anticancer quinones (including anthracycline antibiotics, mitomycin C, mitoxantrone, and menadione) on cyanide-resistant respiration by rat heart myocytes. As demonstrated in Table 2, essentially all the anthracyclines tested significantly increased cardiac oxygen consumption. Only 5-iminodaunorubicin, which has a substituted quinone group that substantially limits oxidation-reduction cycling [24] was incapable of stimulating myocyte oxygen consumption under our experimental conditions. Of the nonanthracyclines examined, both mitomycin C and menadione (2-methyl-1,4 naphthoquinone) stimulated cyanide-resistant respiration significantly, whereas mitoxantrone, at equimolar concentration, proved ineffective (Table 2). These results are consistent with previous investigations suggesting that the mitoxantrone quinone does not produce an active redox cycle in the heart mitochondria or sarcoplasmic reticulum [25]. On the other hand, mitomycin C significantly enhances oxygen consumption by cardiac NADPH:cytochrome P-450 reductase but not by the mitochondrial electron transport chain [26]. Although the effect of menadione on myocyte oxygen consumption has not been examined previously, reduction of molecular oxygen by this compound after treatment of hepatocytes has been described [27].

### 3.2. Effect of Oxygen Radical Scavengers on Doxorubicin-Stimulated Myocyte Oxygen Consumption

To further examine the mechanism(s) underlying the increase in myocyte oxygen consumption produced by doxorubicin, we investigated the effect of various oxygen radical modifying agents on drug-related respiration. Both catalase and acetylated cytochrome c, scavengers of hydrogen peroxide and superoxide anion, respectively, significantly decreased doxorubicin-enhanced, cyanide-resistant myocyte oxygen consumption (Table 3). Heat-inactivated catalase was without effect. These results are of interest in that neither protein would be expected to enter viable myocytes. Thus, it seems likely that at least some extracellular superoxide anion and H_2_O_2_ were present following doxorubicin exposure in these studies. The addition of DTPA, an efficient iron chelator that does not penetrate mammalian cell membranes [28], also did not decrease drug-stimulated oxygen consumption. Since DTPA chelates iron in a form that is unavailable for oxidation-reduction reactions, our findings do not support formation of an extracellular doxorubicin-iron complex as the species responsible for reduction of oxygen in these experiments [29]. Drug-related oxygen consumption was, furthermore, not diminished by DMSO, a potent scavenger of the hydroxyl radical; this was expected because DMSO does not detoxify hydrogen peroxide or superoxide anion under aqueous conditions and because the reaction of DMSO with the hydroxyl radical does not yield molecular oxygen [22].

Finally, although previous investigations have demonstrated that treatment of neonatal cardiac myocytes in culture with the anthracycline daunorubicin significantly increases intracellular DT-diaphorase activity [30], addition of the potent DT-diaphorase inhibitor dicumarol did not change the rate of doxorubicin-stimulated myocyte respiration (Table 3). Additional experiments using 50 *μ*M doxorubicin also failed to reveal any effect of dicumarol on drug-stimulated respiration (data not shown). If DT-diaphorase played an important role in cardiac detoxification by catalyzing the two-electron reduction of the doxorubicin quinone, enzyme inhibition by dicumarol should have increased the rate of oxygen consumption by favoring semiquinone over hydroquinone formation.

### 3.3. Effect of Doxorubicin on Hydrogen Peroxide Production by Heart Myocytes

**Figure 3:**
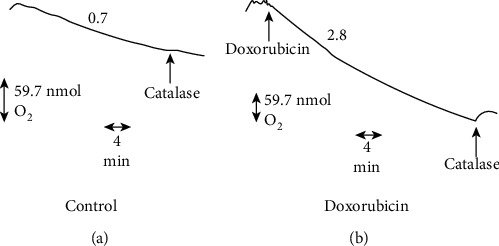
Effect of doxorubicin on cyanide-resistant oxygen consumption and hydrogen peroxide production by rat heart myocytes. Numbers above each experiment indicate oxygen consumption in nmol/min/10^6^ cells. Catalase (arrow, 7500 units) was added through the access slot of the oxygen electrode plunger. (a) The control rate of rat heart myocytes in the presence of 5 mM KCN. (b) The doxorubicin concentration was 400 *μ*M.

As demonstrated in Figure 3, addition of catalase to cyanide-treated myocytes in the presence of doxorubicin released oxygen, indicating that hydrogen peroxide had been formed in these studies. In multiple experiments, identical to the one shown in Figure 3, the rate of hydrogen peroxide formation was 1.30 ± 0.02 nmol/min/10^7^ myocytes (*n* = 4) in the presence of doxorubicin (400 *μ*M). Release of H_2_O_2_ was not detectable in the absence of the drug (*n* = 4, *P* < 0.01). It is important to point out that our studies only measured H_2_O_2_ that leaves the cell and is accessible to exogenous catalase. Thus, these results represent a lower limit estimate of the rate of drug-stimulated H_2_O_2_ formation. Furthermore, because of this methodological limitation, no precise quantitation of the stoichiometry between myocyte oxygen consumption and H_2_O_2_ production is possible under our experimental conditions.

## 4. Discussion

In these experiments, we have demonstrated that a wide range of anthracycline antibiotics, as well as mitomycin C and menadione, are capable of stimulating oxygen consumption by heart myocytes isolated from adult rats. Furthermore, drug-related oxygen consumption is accompanied by the formation of hydrogen peroxide. Hence, our studies confirm that reactive oxygen metabolism by anticancer quinones previously studied with cardiac subcellular fractions or purified enzyme preparations occur in the intact cell. Since doxorubicin significantly increased myocyte oxygen consumption in the presence of rotenone (which is not known to inhibit antioxidant enzyme activity) as well as cyanide and led to the presence of detectable extracellular levels of H_2_O_2_, our investigations suggest that reactive oxygen formation after treatment of cardiac myocytes with doxorubicin exceeds cellular pathways for both enzymatic and nonenzymatic detoxification of oxygen radical metabolites. Hence, it seems possible that drug-related oxygen radical formation *in vivo* could be involved in the cardiac toxicity of the anthracycline quinones.

It has been recognized for several years that the risk of clinical congestive heart failure is increased in patients who are treated with mitomycin C after having previously received doxorubicin [31, 32]. Furthermore, previous studies have demonstrated that mitomycin C increases oxygen consumption and superoxide production by NADPH:cytochrome P-450 reductase from the heart sarcoplasmic reticulum; however, mitomycin C is not actively reduced by the mitochondrial electron transport chain [26]. At equimolar concentrations, in this study, mitomycin C and doxorubicin increased myocyte oxygen consumption by 52% and 56.3%, respectively (Tables 1 and 2). Hence, these experiments suggest that mitomycin C may undergo more limited free radical metabolism than the anthracyclines in intact myocytes. Because of the abundance of mitochondria in the heart, and because of the clinical observation that mitomycin C by itself is seldom a cardiotoxin, it may be speculated that reduction of the anthracycline quinone by the mitochondria plays a predominant role in generating drug-induced free radicals in intact heart cells.

We also found in these experiments that oxygen consumption was not decreased by a potent iron-chelating agent. This finding does not eliminate the possibility that iron may play an important role in the myocardial toxicity of anticancer quinones. Our experiments suggest only that an extracellular doxorubicin-iron complex was unlikely to be the cause of the oxygen radical production that we observed. The role of an intracellular drug-iron complex or of free or protein-bound iron in facilitating the production of the hydroxyl radical or other reactive species has not been examined in these experiments. However, because of the potent oxidizing power of the hydroxyl radical, and studies suggesting that this species is of importance to anthracycline toxicity [33]; further definition of the role of intracellular iron in anthracycline heart toxicity remains of interest.

## 5. Conclusions

In summary, the experiments reported here strongly suggest that adult rat heart myocytes metabolize anticancer quinones to species capable of reducing molecular oxygen. It is noteworthy, furthermore, that generation of reactive oxygen species occurred at anthracycline concentrations that are formed intracellularly following exposure of intact heart cells to this class of drugs [34]. In view of the presence of an extracellular flux of H_2_O_2_ following exposure of rat heart myocytes to doxorubicin, and the recent demonstration of the adverse consequences of a doxorubicin-related free radical cascade on cardiac fibroblasts [15], these experiments suggest, finally, that multiple components of the cardiac matrix, as well as myocyte membranes, may be at risk from anthracycline-induced oxidative stress.

## Figures and Tables

**Table 1:** Requirements for quinone-enhanced oxygen consumption in rat heart myocytes. Oxygen consumption was determined polarographically in a 3 ml reaction system at 37°C that contained 125 mM potassium phosphate buffer, pH 7.4, 140 mM NaCl, 10 mM glucose, and 2 × 10^6^ viable cardiac myocytes.

Reaction system	Oxygen consumption (nmol O_2_/min/2 × 10^6^ cells)
Control	5.35 ± 0.38^a^
+KCN (5 mM)	1.02 ± 0.08^b^
+Rotenone (10 *μ*M)	1.52 ± 0.02^b^
Doxorubicin (400 *μ*M)	
+Rotenone (10 *μ*M)	2.20 ± 0.14^c^
Doxorubicin (400 *μ*M)	
+KCN (5 mM)	5.74 ± 0.28^c^
Doxorubicin (400 *μ*M)	
+KCN (5 mM)	
-Cells	ND^d^

^a^Mean ± S.E. of 3 to 5 experiments. ^b^Significantly different from the control, at *P* < 0.01. ^c^Significantly different from samples containing mitochondrial inhibitors alone, at *P* < 0.01. ^d^N.D. is not detectable.

**Table 2:** Effect of anticancer quinones on cyanide-resistant oxygen consumption by cardiac myocytes. Oxygen consumption was measured with a Clark-type electrode in a 3 ml reaction system at 37°C that contained 125 mM potassium phosphate buffer, pH 7.4, 140 mM NaCl, 10 mM glucose, 5 mM KCN, and 2 × 10^6^ viable cardiac myocytes, with or without drugs.

Drug	Oxygen consumption (nmol O_2_/min/2×10^6^ cells)
None	1.02 ± 0.08^a^
4-Demethoxydaunorubicin	
50 *μ*M	1.82 ± 0.15^b^
400 *μ*M	2.22 ± 0.02^b^
4′-Epidoxorubicin (400 *μ*M)	3.48 ± 0.34^b^
Menogaril (400 *μ*M)	2.57 ± 0.06^b^
4′-Deoxydoxorubicin (400 *μ*M)	2.67 ± 0.53^b^
5-Iminodaunorubicin (400 *μ*M)	1.24 ± 0.24
Mitomycin C	
50 *μ*M	1.08 ± 0.06
400 *μ*M	1.55 ± 0.06^b^
Mitoxantrone (400 *μ*M)	1.39 ± 0.11
Menadione	
10 *μ*M	1.90 ± 0.30^b^
50 *μ*M	4.80 ± 1.20^b^
400 *μ*M	9.37 ± 1.41^b^

^a^Mean ± S.E. of 3 to 5 experiments. ^b^Significantly different from the control, at *P* < 0.05.

**Table 3:** Effect of oxygen radical modifiers on doxorubicin-enhanced, cyanide-resistant oxygen consumption by rat cardiac myocytes. Oxygen consumption was measured with a Clark-type electrode in a 3 ml reaction system at 37°C exactly as described in Table 2.

Reaction system	Oxygen consumption (percent of control)
Control	100^a^
+Catalase (2500 units/ml)	102 ± 8^b^
+Heat-inactivated catalase (2500 units/ml)^c^	134 ± 24
+Acetylated cytochrome c (50 *μ*M)	93 ± 3
+Dicumarol (50 *μ*M)	125 ± 25
Doxorubicin (400 *μ*M)	100^d^
+Catalase (2500 units/ml)	57 ± 1^e^
+Heat-inactivated catalase (2500 units/ml)	103 ± 5
+Acetylated cytochrome c (50 *μ*M)	81 ± 5^e^
+Dicumarol (50 *μ*M)	97 ± 9
+DTPA (100 *μ*M)	99 ± 6
+DMSO (100 mM)	100 ± 3

^a^Control rate of oxygen consumption was 1.00 ± 0.05 nmol/min/2 × 10^6^ myocytes, *n* = 4. ^b^Mean ± S.E. of 3 to 5 experiments. ^c^Catalase was inactivated by autoclaving for 60 min. ^d^Doxorubicin-stimulated, cyanide-resistant oxygen consumption was 6.4 ± 0.6 nmol/min/2 × 10^6^ cells, *n* = 3. ^e^Significantly different from samples containing doxorubicin alone, *P* < 0.05.

## Data Availability

The data used to support the findings of this study are included with the article.

## Abbreviations

DMSO: Dimethyl sulfoxide
DTPA: Diethylenetriamine-pentaacetic acid
SOD: Superoxide dismutase
KCN: Potassium cyanide.
